# Sequential Interventional Management of Osseous Neoplasms via Embolization, Cryoablation, and Osteoplasty

**DOI:** 10.1155/2019/5247837

**Published:** 2019-04-14

**Authors:** Sri Hari Sundararajan, Steven Calamita, Peter Girgis, Gregory Ngo, Srirajkumar Ranganathan, Marisa Giglio, Vyacheslav Gendel, Sharad Goyal, John Nosher, Sudipta Roychowdhury

**Affiliations:** ^1^Department of Radiology, Weill Cornell Medical College, 525 East 68th Street, New York, NY 10065, USA; ^2^Department of Radiology, Dartmouth-Hitchcock Medical Center, One Medical Center Drive, Lebanon, NH 03756, USA; ^3^Department of Radiology, Rutgers-Robert Wood Johnson Medical School, 1 Robert Wood Johnson Place, New Brunswick, NJ 08901, USA; ^4^Northwestern University Feinberg School of Medicine, Chicago, IL, USA; ^5^DePaul University - Lincoln Park Campus, 2400 N Sheffield Ave, Chicago, IL 60614, USA; ^6^Penn State College of Medicine, 500 University Drive, Hershey, PA 17033, USA; ^7^Department of Radiology, Northwell Lenox Hill Hospital, 100 E 77th St, New York, NY 10075, USA; ^8^Rutgers-Cancer Institute of New Jersey, Department of Radiation Oncology, 195 Little Albany St, New Brunswick, NJ 08901, USA

## Abstract

The purpose of this study is to determine if sequential interventional therapy can become a mainstay option in providing palliation from fastidious osseous neoplasms in patients with pain refractory to oral analgesia and radiotherapy. This retrospective monocentric study was approved by our institutional review board. Between July 2012 and August 2014, we reviewed 15 patients (6 women, 9 men; age range of 36-81 years) who underwent embolization followed by cryoablation, with or without osteoplasty. Patient demographics and tumor characteristics, including primary histology and the location of metastasis, were included in our review. Pain intensity at baseline, after radiotherapy, and after sequential interventional therapy was reviewed using the hospital electronic medical record. The use of oral analgesia and procedural complications was also noted. Data was then assessed for normality and a two-tailed Student's* t*-test was performed on mean pain scores for difference phases of treatment. While radiotherapy offers pain relief with a mean pain score of 7.25 ±1.5 (p =<.0001), sequential interventional therapy results in better comfort as demonstrated by a mean pain score of 3.9 ± 2.6 (p=.0015). Moreover, all patients who reported oral analgesic use at presentation reported a decrease in their requirement after sequential interventional therapy. Embolization and cryoablation were performed in all patients, while osteoplasty was indicated in 6 cases. There was no difference in postprocedural pain intensity between patients who required osteoplasty and patients who did not (p = 0.7514). There were no complications observed during treatment. This retrospective study shows that sequential intervention with transarterial embolization, cryoablation, and osteoplasty is both safe and efficacious for bone pain refractory to the current standard of care. We demonstrated that this combination therapy has the potential to become an effective mainstay treatment paradigm in the palliative care of osseous neoplasm to improve quality of life.

## 1. Introduction 

Both primary malignant and metastatic osseous neoplasms are a significant cause of cancer morbidity and mortality [1]. Metastatic osseous neoplasms are more prevalent, with primary tumors most frequently originating from breast, prostate, and kidney cancer. Metastases are associated with fracture, spinal cord compression, and hypercalcemia, with pain as the cardinal symptom affecting up to 80* *% of patients [2]. Both primary and metastatic osseous neoplasms can be difficult to treat, given their ability to alter weight-bearing mechanics and their propensity to invade adjacent neurovascular bundles. Tumor burden and associated pain can prove difficult to manage despite comprehensive medical management due to radiotherapy (RT) resistance, maximization of analgesia, and noncandidacy for surgery. In fact, 20-30% of patients treated with external beam radiation therapy do not experience primary pain relief [3]. Minimally invasive treatment options have been well described and are preferred, particularly in patients with metastatic disease and low 5-year survival rates [4]. Multicenter studies have demonstrated that radiofrequency (RF) ablation is efficacious for pain relief in patients with osseous metastases [5]. However, a significant limitation of RF ablation is the inability of intraprocedural visualization of the ablation zone, thereby increasing the complication rate. Cryoablation allows for greater control of the ablation margin because a low-attenuation ice ball is identifiable on computed tomography (CT) monitoring. This allows cryoablation to be performed in close proximity to critical structures, as observed with spinal metastases. Thus, the efficacy and safety of image-guided percutaneous cryoablation for painful osseous neoplasms have been well established [6, 7]. However, recurrence of disease or incomplete alleviation of symptoms can occur despite the above treatment [4]. Transarterial embolization has been used to decrease hemorrhage associated with both surgical and minimally invasive interventions [8]. In addition, embolization has been used in cases in which ablation would compromise neurovascular structures [9]. We hypothesize that a treatment paradigm of sequential therapy (ST) with arterial embolization, cryoablation, and osteoplasty will demonstrate significant improvement in pain and improved quality of life with minimal complications.

## 2. Materials and Methods 

### 2.1. Study Design

This retrospective monocentric study was approved by the institutional review board. All patients who underwent the paradigm of embolization, cryoablation, and osteoplasty received thorough informed consent at the time of procedure. Consent included explaining the risks versus benefits of undergoing their treatments, as well as collection of images and permission for publishing their deidentified imaging and clinical information. All patients were referred to our interventional radiologists and triaged on the basis of their clinical exam and diagnostic imaging. Fifteen patients' medical records from July 2012 to August 2014 from our institution were reviewed and deemed eligible based on our inclusion criteria. Patient's demographics and tumor characteristics are provided in Table 1. The mean age was 62 and the most common location of metastasis was the sacrum. With the exception of one patient, all subjects underwent prior radiation therapy ranging from one to three cycles. The patient's primary neoplasm, location of metastasis, and whether they underwent RT were noted. Both the initial pain scores and pain scores after RT were documented. Patients who underwent interventional sequential therapy were followed up between one and eight weeks and their pain scores after ST were documented. Analgesia reduction was determined by chart review with any noted reduction qualifying as a positive finding. Complications were assessed by the interventional radiology team both at the time of the procedure and during follow-up.

### 2.2. Inclusion/Exclusion Criteria

Inclusion criteria included men and women of 19-81 years of ages with primary or secondary osseous neoplasms and inoperable tumor burden who have pretreatment CT or magnetic resonance (MR) imaging charactering the extent of disease. Subjects may or may not have undergone treatment of the underlying condition. Patient must have received sequential embolization and cryoablation. Patients may or may not have been treated with osteoplasty. Subjects must have undergone follow-up imaging in one to three months following treatment to assess effects. Exclusion criteria include absence of any of the above.

### 2.3. Methods of Intervention

Cryoablation with preprocedural angiographic embolization was offered as a means of decreasing patient pain. Postablation osteoplasty was offered for improved stabilization in select patients. Informed consent was obtained after explaining the risks and benefits of the procedures, as well as explaining the procedures that would be performed for palliative measures. The discussed risks included bleeding, infection, potential injury to nonpathological bone, and regional organ damage. The patient, prior to pursuing treatment, understood these risks. Data related to the procedures was recorded under an IRB-approved protocol

**Figure 1:**
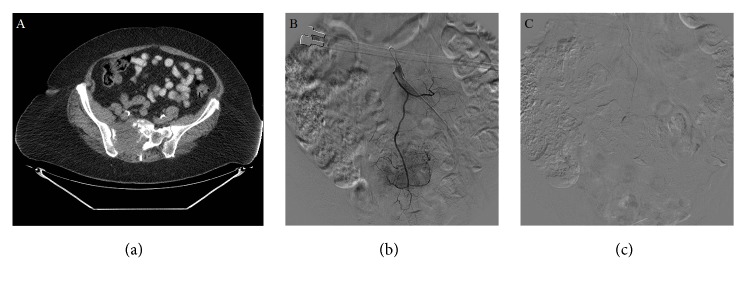
(a) Axial CT demonstrating osseous neoplasm in right sacroiliac wing in 72-year-old female patient. (b) Selective angiography of median sacral artery showing tumor blush and tumor vascularity. (c) Embolization of medical sacral artery with 300 micron PVA particles with diminished vascular supply.

All procedures were performed under general anesthesia with total intravenous anesthesia (TIVA) in conjunction with succinylcholine administered by a trained anesthesiologist. A certified intraoperative neural monitoring specialist was present in the room at all times. A neurologist oversaw monitoring remotely. Cryoablation and osteoplasty typically follow one to two days after embolization. Angiographic embolization was performed prior to cryoablation to decrease tumor perfusion. Vessels were selected based on the location of the neoplasm. The internal and external iliac vessels were targeted for iliac and ischial lesions. The lateral/median sacral, gluteal, and iliolumbar vessels were targeted for sacral lesions. The thoracoacromial and circumflex humeral vessels were targeted for scapular lesions. Sodium Brevital 3 mg (JHP Pharmaceuticals, New Jersey) was injected prior to embolization to assess if changes in recorded transcranial motor evoked potentials (TcMEPs) were visualized. Waveforms remained stable after Brevital injection, following which embolization was performed using 300 to 500 micron polyvinyl alcohol (PVA) particles (Boston Scientific, Massachusetts). Postangiography images showed significantly diminished tumor vascularity (Figure 1).

**Figure 2:**
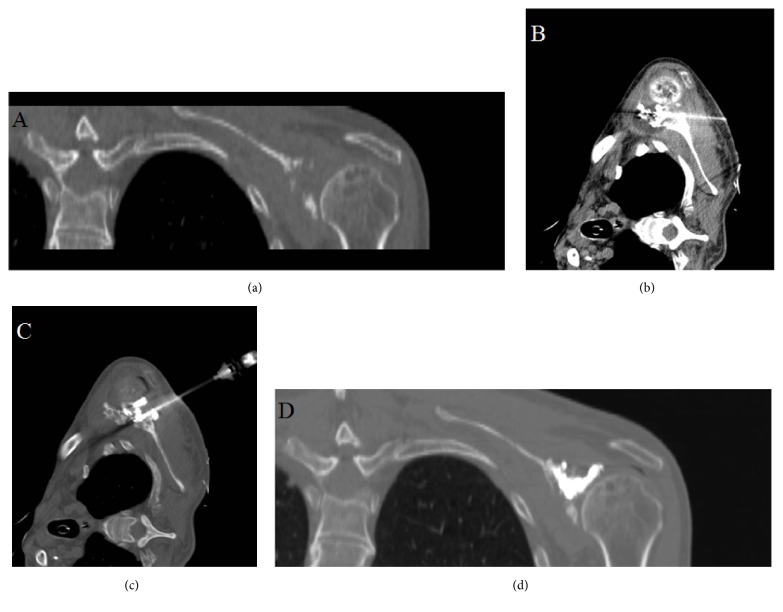
(a) Coronal CT demonstrating osteosarcoma of scapula in 69-year-old male patient. (b) Cryoablation probes inserted through scapula with ovoid intralesional ice ball formation. (c) Axial CT demonstrating cement injection into ablation cavity. (d) Postosteoplasty image illustrating bony reconstruction.

The patients were then brought back within two days to undergo cryoablation. The patients were placed in the appropriate position and prepped and draped in a sterile fashion. Bilateral 10-guage bone biopsy needles serving as introducers were inserted into the osseous lesion. 15 mm cryoablation probes were inserted through each introducer away from adjacent neurovascular structures. Real-time temperature monitoring was accomplished with a temperature probe. Once positioning was verified, a single freeze-thaw-freeze cycle was performed for 10, 8, and 10 minutes, respectively. This resulted in formation of ovoid ice balls within the boundaries of the lesion (Figure 2). While the measured temperature of the cryoablation probe central zone reached -40°C, the temperature probe in surrounding normal bone never decreased below 34°C. Osteoplasty was then performed on the same day with injection of methyl-methacrylate into both 10-guage needles. The lesions were filled with cement under rapid CT guidance to verify no cement extravasation (Figure 2). The needles were subsequently removed and adequate hemostasis was achieved using manual compression. The primary and secondary outcomes were pain evaluation and oral analgesic usage, respectively.

### 2.4. Statistics

This was a single-arm retrospective study in which patients were their own controls. Primary endpoints were pain at baseline, pain after RT, and pain after ST. Pain was measured using the numeric rating scale (NRS-11) [10]. Secondary endpoints were the percentage of patients with a reduction in analgesic use following treatment and any reported complications. Data were tested for Gaussian distribution with D'Agostino-Pearson omnibus normality test. A two-tailed Student's* t*-test was performed comparing mean pain scores at baseline and after RT and mean pain scores after RT and after ST. For patients with no reported post-ST pain scores, their baseline and post-RT scores were still included in our analysis. We also assessed mean pain scores between patients who received osteoplasty and patients who did not receive osteoplasty using Mann-Whitney* U* test. Data are presented as mean ± SD and differences were considered statistically significant at p<0.05. Statistical tests were performed using GraphPad Prism 7.0 software.

## 3. Results

Between July 2012 and August 2014, nine patients underwent embolization, cryoablation, and osteoplasty. Six patients underwent embolization and cryoablation only. Fourteen patients were treated with RT prior. Postprocedural pain was assessed between one and eight weeks, with an average of 3 weeks. All patients had documented baseline and post-RT pain scores. Twelve of fifteen patients (80%) had documented NRS-11 pain score at follow-up. Technical success was achieved in all procedures. Symptomatic neoplasms were fully embolized with stasis achieved in tumor feeding vessels. Cryoablation was performed with visualization of the ice ball on CT. No complications occurred at any point during the embolization, cryoablation, or osteoplasty.

**Figure 3:**
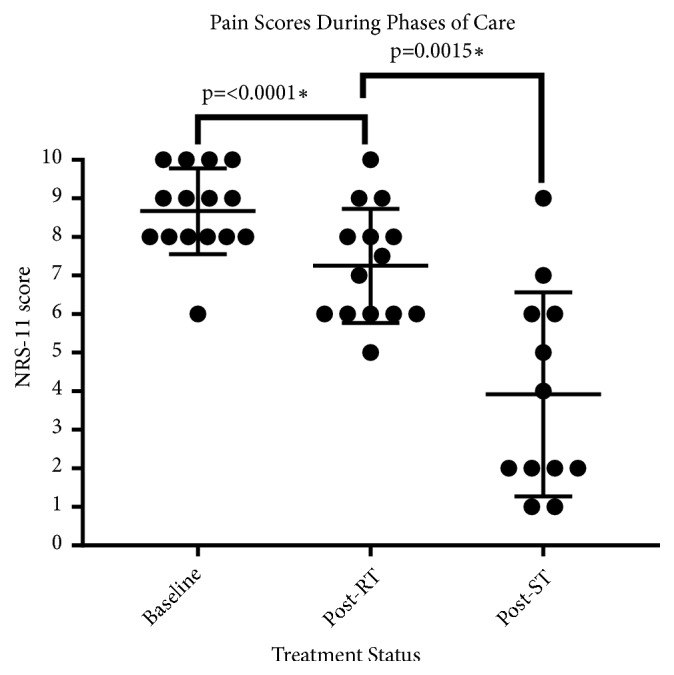
Patient reported NRS-11 pain scores throughout phases of care. Mean pain at baseline was 8.7 ± 1.1. Mean pain after RT was 7.25 ±1.5 (p =<0.0001). Mean pain after ST was 3.9 ± 2.6 (p=0.0015). Statistical significance was set to p < 0.05.

**Figure 4:**
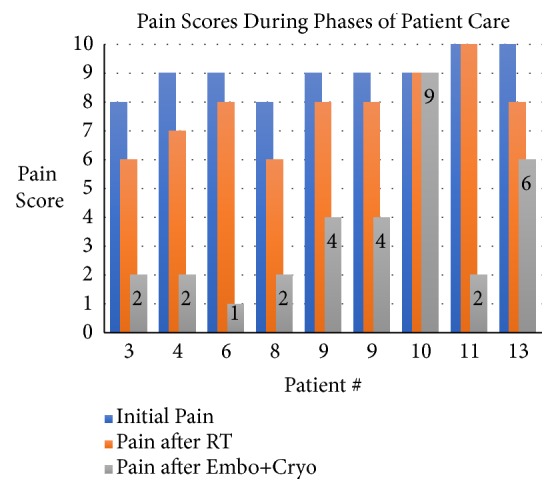
Patient #3 (unknown primary), patient #4 (lung primary), patient #6 (endometrial primary), patient #8 (renal primary), patient #9 (pancreatic primary), patient #10 (osteosarcoma primary), patient #11 (lung primary), and patient #13 (urothelial primary).

Figure 3 shows pain scores charted throughout the different phases of care for each patient. Treatment correlates significantly with primary pain relief. Mean pain at baseline was 8.7 ± 1.1. Patients reported pain reduction after RT with mean pain scores of 7.25 ±1.5 (p =<0.0001). After ST, mean pain scores were further reduced to 3.9 ± 2.6 (p=0.0015). All patients who were using oral analgesics reported decrease use (13/13, 100%). Osteoplasty was performed in seven cases when indicated. Patients without large bony defects and who at the time were judged not to benefit from the procedure did not undergo osteoplasty.  Figure 4 shows pain scores in a color-coded bar graph for a subset of patients with different primary neoplasms treated with ST.

There was no difference in postprocedural pain scores between patients who required osteoplasty and patients who did not (p = 0.7514). One patient did not report postprocedural pain improvements. This patient did, however, report lifestyle improvements with the ability to lay on the side of the tumor.

## 4. Discussion

Minimally invasive treatment options for both primary and metastatic osseous neoplasms have been developed and tested over the past decade [3]. Studies have assessed the efficacy of various ablative techniques, embolization, and osteoplasty as standalone treatments. Yet, when the condition to be treated is multifactorial, so too should the treatment regimen. As no single procedure is uniformly superior to another, incorporation of several effective treatment options based on the clinical symptomatology, histopathological grading, and staging and the radiologic-defined anatomic extent of disease may provide justification for the proposed treatment paradigm. For example, it has been established that transarterial embolization before cryoablation reduces postprocedural related hemorrhage [11, 12]. In addition, embolization allows for the treatment of tumors located near neurovascular structures in which the full margin cannot be covered by cryoablation alone. Other ablative procedures such as radiofrequency ablation have also been studied and proven to be efficacious. However, due to lack of visualization of the ablation zone, it may be less useful near high-risk neurovascular structures. Given this limitation, cryoablation has become a more important procedure in part due to better control of the ablation zone [13]. Mechanistically, cryoablation achieves tissue death via rapid freezing which results in intracellular ice formation and cellular destruction as osmotic pressures rise and cells dehydrate. Recent research suggests that, in addition to physical cell death, cryoablation results in the activation of apoptosis cascades and the modulation of host immune system function to bypass cancer cell's defensive capabilities [14]. Therefore, cryoablation may have an anticancer effect due to its effects on the microenvironment. Finally, osteoplasty may be performed, particularly in cases of osteolytic disease. Osteoplasty has a role as the ablative procedures do not provide support to the bony structure and may result in incomplete pain relief and reduced stability [15].

Clinically, bone pain is managed with a spectrum of analgesics beginning with nonsteroidal anti-inflammatory drugs and culminating in opiate therapy. Adjuvant therapies such as RT and nerve blocks are also common. Studies suggest that the pathophysiology of bone pain is multifactorial. Cancer cells and the associated stroma express receptor activator of nuclear factor *κ*-B ligand (RANKL) which interacts with osteoclast expressed RANK resulting in bone resorption. This process involves an acidic area adjacent to the bone that results in upregulation of specific ion channels, such as transient receptor potential cation channel subfamily V member 1 (TrpV1) [16], which results in cancer bone pain through sensitization and activation of nerve fibers. Endogenous substances such as formaldehyde and osteoblast derived insulin-like growth factor I have been shown to activate TrpV1 receptors as well. Additionally, osteoclastic resorption distorts bone architecture resulting in microfractures which also contribute to pain. In addition to RANKL expression, tumor cells and stroma can directly secrete prostaglandins, proteases, and endothelins which have also been associated with increased pain perception [17]. Therefore, the aforementioned antitumor effects of cryoablation may be further explained by decreasing the production of algogenic substances occurring after tumor death.

Our study results suggest that sequential embolization, cryoablation, and osteoplasty are effective and yield a statistically significant improvement in pain control without complications. This subjective reduction in reported pain scores was confirmed with a uniform reduction in postprocedural oral analgesics in all patients. We have demonstrated that these procedures are safe and efficient and have a role in patients who are symptomatic despite both maximal medical treatment and RT. The pain relief observed is primarily related to cryoablation. Embolization prior to cryoablation provides hemostatic control and allows for targeted therapy of extraosseous disease and osseous sites not amenable to safe cryoablation. Limitations to this study are primarily related to a retrospective design. We were only able to identify 15 patients who underwent ST. This small sample size limited our statistical power. In addition, the histology studied may not be representative of the general population. For example, the primary tumor dictates the characteristics of the metastasis (osteolytic versus osteoblastic), which can impact treatment decisions and outcomes. In addition, we had to rely on chart review to obtain postprocedural pain scores, which were not always quantified. Further studies should be prospectively designed and include a larger sample size with more varied pathology and longer patient follow-up to assess recurrence.

## 5. Conclusions

In summary, ST of painful osseous neoplasms with transarterial embolization, cryoablation, and osteoplasty results in a dramatic reduction in reported pain and analgesic use without complications. We demonstrated that this combination has the potential to become a mainstay paradigm in the palliative care of osseous neoplasm for patients otherwise resistant to radiotherapy. We suggest that a multidisciplinary team implementing optimal medication, RT, and interventional procedures can provide comprehensive management of these patients and improve their quality of life.

## Figures and Tables

**Table 1:** Patient and tumor characteristics.

Characteristic	Datum
No. of patients (female/male) n=15	6/9
Mean age, y (+/- SD)	62 (13)
Range	36-81
Previous RT	14/15 (93%)
Oral analgesics at presentation	13/15 (87%)
Tumor type histology (n=15)	
Lung	4 (26%)
Urothelial	3 (20%)
Renal	2 (13%)
Pancreas	1 (7%)
Breast	1 (7%)
Osteosarcoma	1 (7%)
Endometrial	1 (7%)
Colorectal	1 (7%)
Other*∗*	1 (7%)
Tumor Location (n=16)	
Sacrum	8 (50%)
Scapula	3 (19%)
Ilium	3 (19%)
Ischium	3 (19%)
Embolization and cryoablation only	6/15 (40%)
Embolization, cryoablation, and osteoplasty	9/15 (60%)
Patients with one metastasis†	14/15 (93%)
Patients with two metastases†	1/15 (7%)

*∗*Primary adenocarcinoma of unknown origin.

† considers only metastases that were treated with ST, not total metastases.

## Data Availability

The data used to support the findings of this study are included within the article and the data sources are available from the corresponding author upon request.
